# O.4.4-11 Must we tell people to be less active to save the planet? The dilemma of physical activity promotion from a holistic health perspective

**DOI:** 10.1093/eurpub/ckad133.208

**Published:** 2023-09-11

**Authors:** Peter Gelius, Maike Till, Sven Messing, Antonina Tcymbal, Karim Abu-Omar

**Affiliations:** Friedrich-Alexander-Universität Erlangen-Nürnberg, Germany; peter.gelius@fau.de; Friedrich-Alexander-Universität Erlangen-Nürnberg, Germany; peter.gelius@fau.de; Friedrich-Alexander-Universität Erlangen-Nürnberg, Germany; peter.gelius@fau.de; Friedrich-Alexander-Universität Erlangen-Nürnberg, Germany; peter.gelius@fau.de; Friedrich-Alexander-Universität Erlangen-Nürnberg, Germany; peter.gelius@fau.de

## Abstract

**Purpose:**

The emerging climate crisis poses major challenges for the planet and leaves us to critically reconsider our physical activity (PA) behavior and its potentially negative effects on the environment and climate. Until now, both PA guidelines for individuals and PA promotion policies follow a logic of “more is better”. We argue that this is because PA promotion is still largely based on an individualistic concept of health, while broader concepts such as Global Health, One Health, and Planetary Health are largely ignored. We aim to provide some initial input and guidance on how PA promotion would have to change if it were based on the different concepts.

**Methods:**

Using a qualitative analysis of relevant approaches, we developed a framework to distinguish three classes of health perspectives and their implications for PA promotion. We analyzed their current application in PA promotion and point out potential omissions and shortcomings.

The findings were used to critically review the pros and cons of a potential paradigm shift for PA promotion and its implications for the field.

**Results:**

We identified a total of nine health perspectives, which can be categorized into three main classes ranging from narrow to a wide: 1) individual perspectives, 2) population perspectives and 3) holistic perspectives. While there are numerous types of PA for individuals to choose from to improve health from perspectives purely focused on the individual, broader perspectives increasingly limit the range of options: The more aspects (other people, foreign countries, animals, the global climate) are considered, the more physical activities may have to be considered as “unhealthy”. If such perspectives are accepted, this may have far-reaching implications for future PA recommendations, programs and policies.

**Conclusions:**

Taking on more holistic health perspectives on PA promotion would have far-reaching implications for the field and leave research, policy and civil society with major challenges. In particular, this may imply a need to include animal and environmental health in new PA guidelines for individuals and public policy, and to work towards a “modal shift” towards promoting more environmentally and climate friendly forms of PA and sport.

